# Supplementary material for: Role and mechanism of NCAPD3 in promoting malignant behaviors in gastric cancer
Source: Front Pharmacol. 2024 Apr 22;15:1341039. doi: 10.3389/fphar.2024.1341039 (PMC11070777; doi:10.3389/fphar.2024.1341039)
Supplement: Supplementary file 11 [file DataSheet2.ZIP › GSEA/Oncogenic signature/my_analysis.Gsea.1599461962066/GCNP_SHH_UP_LATE.V1_DN.html]

Details for gene set GCNP\_SHH\_UP\_LATE.V1\_DN[GSEA]

|  || Dataset | filtered\_dataset.sample\_info.cls#WT\_versus\_NCAPD3\_MUT |
| Phenotype | sample\_info.cls#WT\_versus\_NCAPD3\_MUT |
| Upregulated in class | WT |
| GeneSet | GCNP\_SHH\_UP\_LATE.V1\_DN |
| Enrichment Score (ES) | 0.38436043 |
| Normalized Enrichment Score (NES) | 1.5213091 |
| Nominal p-value | 0.068627454 |
| FDR q-value | 0.20378803 |
| FWER p-Value | 0.962 |
Table: GSEA Results Summary

  

Fig 1: Enrichment plot: GCNP\_SHH\_UP\_LATE.V1\_DN      
 Profile of the Running ES Score & Positions of GeneSet Members on the Rank Ordered List

  

| SYMBOL | TITLE | RANK IN GENE LIST | RANK METRIC SCORE | RUNNING ES | CORE ENRICHMENT || 1 | 119467 | CLRN3 | 42 | 0.944 | 0.0800 | Yes |
| 2 | 23054 | NCOA6 | 77 | 0.838 | 0.1534 | Yes |
| 3 | 6573 | SLC19A1 | 231 | 0.650 | 0.1195 | Yes |
| 4 | 81889 | FAHD1 | 299 | 0.592 | 0.1404 | Yes |
| 5 | 23216 | TBC1D1 | 355 | 0.563 | 0.1667 | Yes |
| 6 | 5930 | RBBP6 | 390 | 0.543 | 0.2056 | Yes |
| 7 | 741 | ZNHIT2 | 453 | 0.503 | 0.2198 | Yes |
| 8 | 9690 | UBE3C | 470 | 0.493 | 0.2658 | Yes |
| 9 | 64866 | CDCP1 | 515 | 0.466 | 0.2886 | Yes |
| 10 | 7447 | VSNL1 | 521 | 0.461 | 0.3387 | Yes |
| 11 | 3836 | KPNA1 | 548 | 0.448 | 0.3724 | Yes |
| 12 | 26100 | WIPI2 | 601 | 0.423 | 0.3844 | Yes |
| 13 | 6919 | TCEA2 | 712 | 0.368 | 0.3484 | No |
| 14 | 6303 | SAT1 | 850 | -0.233 | 0.2774 | No |
| 15 | 3918 | LAMC2 | 1088 | -0.466 | 0.1618 | No |
| 16 | 5795 | PTPRJ | 1210 | -0.586 | 0.1434 | No |
Table: GSEA details [plain text format]

  

Fig 2: GCNP\_SHH\_UP\_LATE.V1\_DN      
 Blue-Pink O' Gram in the Space of the Analyzed GeneSet

  

Fig 3: GCNP\_SHH\_UP\_LATE.V1\_DN: Random ES distribution      
 Gene set null distribution of ES for **GCNP\_SHH\_UP\_LATE.V1\_DN**
